# Supplementary material for: Employing a MEMS plasma switch for conditioning high-voltage kinetic energy harvesters
Source: Nat Commun. 2020 Jun 26;11:3221. doi: 10.1038/s41467-020-17019-5 (PMC7319968; doi:10.1038/s41467-020-17019-5)
Supplement: Supplementary file 1 — Supplementary Information [file 41467_2020_17019_MOESM1_ESM.docx]

**Supplementary Information for**

**Employing a MEMS plasma switch for conditioning high-voltage kinetic energy harvesters**

Hemin Zhang^1,2^, Frédéric Marty^1^, Xin Xia^3^, Yunlong Zi^3^, Tarik Bourouina^1^,
Dimitri Galayko^4^* and Philippe Basset^1^*

^1^ESYCOM, Univ Gustave Eiffel, CNRS, CNAM, ESIEE Paris, F‐77454 Marne‐la‐Vallée, France.
^2^Now at Department of Engineering, The Nanoscience Centre, University of Cambridge, Cambridge CB3 0FF, United
 Kingdom.
^3^The Chinese University of Hong Kong, Shatin, N.T., Hong Kong SAR, China.
^4^ Sorbonne Université, LIP6, France.
*Correspondence to: philippe.basset@esiee.fr; dimitri.galayko@sorbonne‐universite.fr

**Supplementary Note 1**

**Working principle of the Bennet Doubler**

The derivations here are used for evaluating the law of the operation of the electrical state of the Bennet’s doubler-based conditioning circuits, powered by a triboelectric nanogenerator. In order to describe the operation of the circuit in the steady state, we evaluate the *i^th^* cycle which, by convention, starts when the variable capacitance is maximum, $\text{C}_{\text{TENG}}\text{ }\text{=}{\text{ }\text{C}}_{\text{max}}$. The parameters are listed in Supplementary Table 1.

We initially assume that the voltage across the three capacitors $\text{C}_{\text{ref}}$, $\text{C}_{\text{buf}}$, and $\text{C}_{\text{TENG}}$ is the same and equal to $\text{V}_{\bar{\text{i}}}$ when starting from point A in Supplementary Figure 2b, at where the TENG has the maximum capacitance of ${\text{ }\text{C}}_{\text{max}};$ D1, D 2and D3 are all OFF; capacitors are in parallel and have the same voltages. The charges in the TENG is $\text{Q}\text{=}\text{V}_{\bar{\text{i}}}\text{C}_{\text{max}}$, until the voltage across the TENG ($V_{\mathrm{TENG}}$) is higher than 2$\text{V}_{\bar{\text{i}}}$, or $\text{C}_{\text{TENG}}$ decreases to $2C_{\min}$ (point B in Fig. 2b). D2 becomes ON and the equivalent circuit is shown in Supplementary Figre 1a, and the capacitors of $\text{C}_{\text{ref}}$ and $\text{C}_{\text{buf}}$ get in series having an equivalent capacitance of $\text{C}_{\text{eq}}\text{=1/(1/}\text{C}_{\text{ref}}\text{+1/}\text{C}_{\text{buf}}\text{)}$. Thus, at the end of the trajectory “*C*_max_🡪*C*_min_” at the point D, the generated charges by the TENG at the 1^st^ phase of *i^th^* cycle can be written as:

| $\text{∆}\text{Q}_{\text{gi}}\text{=(}\text{V}_{\bar{\text{i}}}\text{ + }\text{V}_{\text{TE}}\text{)}\text{C}_{\text{max}}\text{-}(\text{V}_{\text{TENG-}\underline{\text{i}}}\text{+}\text{V}_{\text{TE}}\text{)}\text{C}_{\text{min}}$ | (1) |
| --- | --- |

where $\text{V}_{\text{TENG}\text{-}\underline{\text{i}}}$ is the voltage at point D when $\text{C}_{\text{TENG}}=\text{C}_{\text{min}}$. Note that during the segment B🡪D, all capacitors are in series and this charge $\text{∆}\text{Q}_{\text{gi}}$ is added to the charges of the both capacitors $C_{\mathrm{buf}}$ and $C_{\mathrm{ref}}$ which preexisted at the point A, $\text{V}_{\bar{\text{i}}}\text{C}_{\text{buf}}$ and $\text{V}_{\bar{\text{i}}}\text{C}_{\mathrm{ref}}$ respectively. The voltage $\text{V}_{\text{TEN}\text{G}_{\underline{\text{i}}}}$ at the point D is a sum of the voltages of these two capacitors:

| $\text{V}_{\text{TEN}\text{G}_{\underline{\text{i}}}}\text{=}\frac{\text{C}_{\text{ref}}\text{V}_{\bar{\text{i}}}\text{ }\text{+}\text{ }\text{∆}\text{Q}_{\text{gi}}}{\text{C}_{\text{ref}}}\text{+}\frac{\text{C}_{\text{buf}}\text{V}_{\bar{\text{i}}}\text{ }\text{+}\text{ }\text{∆}\text{Q}_{\text{gi}}}{\text{C}_{\text{buf}}}\text{=2}\text{V}_{\bar{\text{i}}}\text{ }\text{+}\frac{\text{∆}\text{Q}_{\text{gi}}}{\text{C}_{\text{eq}}}$ | (2) |
| --- | --- |

Thus we obtain:

| $\text{V}_{\text{TEN}\text{G}_{\underline{\text{i}}}}\text{=}\frac{\text{V}_{\bar{\text{i}}}\text{(}\text{C}_{\text{max}}\text{+2}\text{C}_{\text{eq}}\text{)+}\text{V}_{\text{TE}}\text{(}\text{C}_{\text{max}}\text{-}\text{C}_{\text{min}}\text{)}}{\text{C}_{\text{min}}\text{+}\text{C}_{\text{eq}}}\text{V}_{\bar{\text{i}}}$ | (3) |
| --- | --- |

And the transferred charges is rewritten as:

| $\text{∆}\text{Q}_{\text{gi}}\text{=}\frac{\text{V}_{\bar{\text{i}}}\text{C}_{\text{eq}}\text{(}\text{C}_{\text{max}}\text{-2}\text{C}_{\text{min}}\text{)+}\text{V}_{\text{TE}}\text{C}_{\text{eq}}\text{(}\text{C}_{\text{max}}\text{-}\text{C}_{\text{min}}\text{)}}{\text{C}_{\text{min}}\text{+}\text{C}_{\text{eq}}}$ | (4) |
| --- | --- |

In the other phase of $C_{\mathrm{TENG}}$ increasing from $\text{C}_{\text{min}}$ to $\text{C}_{\text{max}}$, starting from point D in Fig. 2b, all the three diodes are OFF and voltage across the TENG is dropping until point E where D1 and D3 are ON, indicating that $\text{C}_{\text{ref}}$ and $\text{C}_{\text{buf}}$ are in parallel as shown in Supplementary Figure 1b. During the segment E🡪A, the fixed capacitors give to $\text{C}_{\text{TENG}}$ some charge, so that at the point A the charge balance between the points E and A can be written as:

| $\text{V}_{\bar{\text{i}}}\left( \text{C}_{\text{ref}}\text{+}\text{C}_{\text{buf}} \right)\text{+(}{\text{V}_{\bar{\text{i}}}\text{+}\text{V}}_{\text{TE}}\text{)}\text{C}_{\text{max}}\text{+}\text{∆}\text{Q}_{\text{gi}}\text{=}\text{V}_{\bar{\text{i+1}}}\left( \text{C}_{\text{ref}}\text{+}\text{C}_{\text{buf}} \right)\text{+(}{\text{V}_{\bar{\text{i+1}}}\text{+}\text{V}}_{\text{TE}}\text{)}\text{C}_{\text{max}}$ | (5) |
| --- | --- |

Note that between the states A and E, the TENG capacitor gives $\text{∆}\text{Q}_{\text{gi}}$, while each fixed capacitor receives $\text{∆}\text{Q}_{\text{gi}}$, so that we add only $\text{∆}\text{Q}_{\text{gi}}$ to the charges the capacitors had initially at the state A. Note, that this is the key of the operation of the Bennet’s doubler: after one cycle of operation, the overall charge of in the capacitive tank increase thanks to the alternation between series and parallel topology for positive and negative current respectively.

Substituting Supplementary Equation 4 into the last expression of Supplementary Equation 5, we obtain:

| $\text{V}_{\bar{\text{i+1}}}\text{=}\text{V}_{\bar{\text{i}}}\left[ \text{1+}\frac{\text{C}_{\text{eq}}\text{(}\text{C}_{\text{max}}\text{-2}\text{C}_{\text{min}}\text{)}}{\text{(}\text{C}_{\text{min}}\text{+}\text{C}_{\text{eq}}\text{)(}\text{C}_{\text{max}}\text{+}\text{C}_{\text{ref}}\text{+}\text{C}_{\text{buf}}\text{)}} \right]\text{+}\text{V}_{\text{TE}}\frac{\text{C}_{\text{eq}}\text{(}\text{C}_{\text{max}}\text{-}\text{C}_{\text{min}}\text{)}}{\text{(}\text{C}_{\text{min}}\text{+}\text{C}_{\text{eq}}\text{)(}\text{C}_{\text{max}}\text{+}\text{C}_{\text{ref}}\text{+}\text{C}_{\text{buf}}\text{)}}$ | (6) |
| --- | --- |

From Supplementray Equation 6, we can see that as far as $\text{C}_{\text{max}}\text{>}\text{ }\text{2}\text{C}_{\text{min}}$, at the starting of the (*i+*1)*^th^* cycle the voltage and charges are both higher compared to the *i^th^* cycle. The obtained recurrent equation is known as geometric-arithmetic progression. If at *i*=0 the TENG voltage is $V_{0}$, the TENG voltage at ith cycle is given by:

| $\text{V}_{\bar{\text{i}}}\text{=}\left[ \text{1+}\frac{\text{C}_{\text{eq}}\text{(}\text{C}_{\text{max}}\text{-2}\text{C}_{\text{min}}\text{)}}{\text{(}\text{C}_{\text{min}}\text{+}\text{C}_{\text{eq}}\text{)(}\text{C}_{\text{max}}\text{+}\text{C}_{\text{ref}}\text{+}\text{C}_{\text{buf}}\text{)}} \right]^{\text{i}}\left( \text{V}_{\text{0}}\text{+}\text{V}_{\text{TE}}\frac{\text{C}_{\text{max}}\text{-}\text{C}_{\text{min}}}{\text{C}_{\text{max}}\text{-2}\text{C}_{\text{min}}} \right)\text{-}\text{V}_{\text{TE}}\frac{\text{C}_{\text{max}}\text{-}\text{C}_{\text{min}}}{\text{C}_{\text{max}}\text{-2}\text{C}_{\text{min}}}$ | (7) |
| --- | --- |

Finally, the total energy delivered to the network of three capacitors at the beginning of each cycle is found as:

| $\text{∆}\text{E}_{\bar{\text{i}}}\text{ }\text{=}\frac{\text{1}}{\text{2}}\text{(}\text{C}_{\text{max}}\text{+}\text{C}_{\text{ref}}\text{+}\text{C}_{\text{buf}}\text{)}\text{[}\text{V}_{\bar{\text{i+1}}}^{\text{2}} \text{-}{\text{ }\text{V}}_{\bar{\text{i}}}^{\text{2}}\text{]}\text{, }\text{i}\text{≥1}$ | (8) |
| --- | --- |

The gained energy is distributed over the three capacitors. If one of the capacitors $\text{C}_{\text{ref}}$, $\text{C}_{\text{buf}}$ and $\text{C}_{\text{TENG}}$ dominates the others, virtually all the harvested energy is stored in this capacitor, thus we set the value of $\text{C}_{\text{buf}}\text{>}\text{C}_{\text{ref}}\text{>}\text{C}_{\text{TENG}}$. The parameters used in the LTspice model are listed in the following Supplementary Table 1. Note that the proposed analysis is also valid for capacitive transducers biased by an electret layer.

**Supplementary Note 2**

**Theoretical analysis of the switch with movable electrodes**

When combing the principles of plasma discharge and electrostatic pulling to develop a switch, several complicated cases will occur with different applied voltages. In this section, theoretical analysis is given to explore the relations between the ON-voltage and the hysteresis loop.

First of all, we should claim clearly that there are two gaps that are critical in our switch, i.e. the comb fingers’ gap ($d$), and the gap between anode and cathode ($\text{g}$). The comb fingers are the actual drive sources of the switch movements. Comb drives consist of stationary and suspended moving fingers. If a voltage difference is applied between them, the electrostatic force of the fringe fields pulls the anode close to the cathode. As shown in Supplementary Figure 7a, the actuation capacitance of n pairs of comb fingers can be written as:

| $\text{C}\text{=}\text{2n}\frac{\text{ε}_{\text{0}}\text{(l+x)h}}{\text{d}}\text{+}\text{n}\frac{\text{ε}_{\text{0}}\text{wh}}{\text{(l-x)}}$ | (9) |
| --- | --- |

where $l$ is the overlap, $d$ the gap between fingers, $w$ the width of a finger, $h$ the height of the device layer, $x$ the displacement and $\varepsilon_{0}$ the permittivity in air. In the case of voltage control the lateral electrostatic force in the moving direction is equal to the negative derivative of the electrostatic energy^1^ with respect to *x*:

| $\text{F}_{\text{elec}}\text{=}\frac{\text{1}}{\text{2}}\frac{\text{∂C}}{\text{∂x}}\text{V}^{\text{2}}\text{≈}\text{n}\frac{\text{ε}_{\text{0}}\text{h}}{\text{d}}\text{V}^{\text{2}}\text{-}\frac{\text{n}\text{ε}_{\text{0}}\text{wh}\text{V}^{\text{2}}}{{\text{2}\left( \text{l}\text{-}\text{x} \right)}^{\text{2}}}\text{=}\frac{\text{n}\text{ε}_{\text{0}}\text{h}\text{V}^{\text{2}}}{\text{2}}\text{(}\frac{\text{2}}{\text{d}}\text{-}\frac{\text{w}}{\left( \text{l}\text{-}\text{x} \right)^{\text{2}}}\text{)}$ | (10) |
| --- | --- |

The electrostatic force is acting on the spring where the anode is connected. In our design, $\text{d}\text{ }\text{=}\text{ }\text{4}\text{ }\text{μm}$, $\text{w}\text{ }\text{=4 μm}$, $\text{h}\text{ }\text{=}\text{ }\text{40 μm}$, $\text{l}\text{ }\text{=}\text{ }\text{20 μm}$, thus in the displacement and force expression, the high order term can be ignored and the deflection *x* is given by:

| $\text{x}\text{=}\frac{\text{F}_{\text{e}\text{lec}}}{\text{k}_{\text{eff}}}\text{≈}\frac{\text{n}\text{ε}_{\text{0}}\text{h}\text{V}^{\text{2}}}{\text{d}\text{k}_{\text{eff}}}$ | (11) |
| --- | --- |

The gap between anode and cathode is decreasing as the increase of *x*. As the breakdown voltage is a function of the gap, thus the breakdown voltage is varying with the voltage across *C*_buf_ as well. The Paschen’s Law curve here should not be a classical one, but has a dynamic calibration. The normal Paschen’s Law equation^2^ is:

| $\text{V}_{\text{b}}\text{=}\frac{\text{Bp}\text{g}_{\text{0}}}{\ln\text{(}\text{Ap}\text{g}_{\text{0}}\text{)}\text{-ln(ln(}\frac{\text{1}}{\text{ }\text{γ}}\text{+1))}}$ | (12) |
| --- | --- |

where $\text{g}_{\text{0}}$ is the gap between anode and cathode, and here is set as (6-*x*) μm, (9-*x*) μm, and (12-*x*) μm; $\text{p}$ is the operation pressure; $\text{γ}$ is the gas composition and *A* is the saturation ionization *B* is a constant related to the excitation and ionization energies. The comb finger overlap is designed as 20 μm, much larger than $\text{g}_{\text{0}}$. When considering the deflection of the anode, the fixed equation is:

| $\text{V}_{\text{breakdown}}\text{=}\frac{\text{Bp}\text{(}\text{g}_{\text{0}}\text{-}\text{x}\text{)}}{\ln\text{(}\text{Ap}\text{(}\text{g}_{\text{0}}\text{-}\text{x}\text{))}\text{-ln(ln(}\frac{\text{1}}{\text{γ}}\text{+1))}}$ | (13) |
| --- | --- |

| Parameters | Meaning | Values in simulation |
| --- | --- | --- |
| $\text{C}_{\text{max}}$ | Maximum capacitance of the TENG | 500 pF |
| $\text{C}_{\text{min}}$ | Minimum capacitance of the TENG | 150 pF |
| $\text{V}_{\bar{\text{i}}}$ | Voltage across the TENG at the start of the *i^th^* cycle | --- |
| $\text{V}_{\bar{\boldsymbol{i+1}}}$ | Voltage across the TENG at the start of the (*i+*1)*^th^* cycle | --- |
| $\text{C}_{\text{ref}}$ | Capacitance of $\text{C}_{\text{ref}}$ | 1 nF |
| $\text{C}_{\text{buf}}$ | Capacitance of $\text{C}_{\text{buf}}$ | 4.7 nF |
| Fre | Mechanical actuation frequency | 5 Hz |
| $\text{σ}$ | Surface charge density | 60 μC/m^2^ |

**Supplementary Table 1.** Definition of the parameters for the Bennet doubler in the simulation

**Supplementary Figure 1**. The equivalent circuits of the Bennet doubler in the phase of $\text{C}_{\text{TENG}}$ decreasing from $\text{C}_{\text{max}}$ to $\text{C}_{\text{min}}$ **(a)** and in the phase of $\text{C}_{\text{TENG}}$ increasing from $\text{C}_{\text{min}}$ to $\text{C}_{\text{max}}$ **(b)**.

**Supplementary Figure 2. The experimentally measured voltage across the TENG**. **(a)** Raw measured data. **(b)** Measured data after applying a 40 Hz low-pass filter. **(c)** Low-pass filtered data with a 50%-percentile smoothing of 65 points. In the raw data of the measure voltage, we observe a peak at each front edge followed by a damped oscillation at 50 Hz seeing from the inset figure in Supplementary Figure 2(a). The 50 Hz oscillation comes from the surrounding electromagnetic noise. The peak is directly induced by the setup we used for measuring the high-voltage, i.e. a 1/71 resistive divider made of 2 resistors of 70 GΩ and 1 GΩ. The voltage peak comes from the parasitic capacitance of the 70 GΩ resistor which has a low impedance compared to the resistance with this voltage having such a high slope. Source data are provided as a Source Data file.

**Supplementary Figure 3**. QV cycles of the TENG at different operation cycles (time) with a Bennet doubler as the conditioning circuit. The area of the closed shape indicates the effectively harvested energy per cycle. The mechanical cycle numbers are marked in the figure. A considerable increase of the harvested energy per cycle is observed as the Voltage across the TENG increases. The closed shape is not exactly a rectangular as the simulation because of the low sample rate of the picometer when recording the current and also the inner resistance of the TENG. Source data are provided as a Source Data file.





**Supplementary Figure 4**. Current through the TENG using a full-wave rectifier to charge a capacitor of 4.7 nF. Source data are provided as a Source Data file.


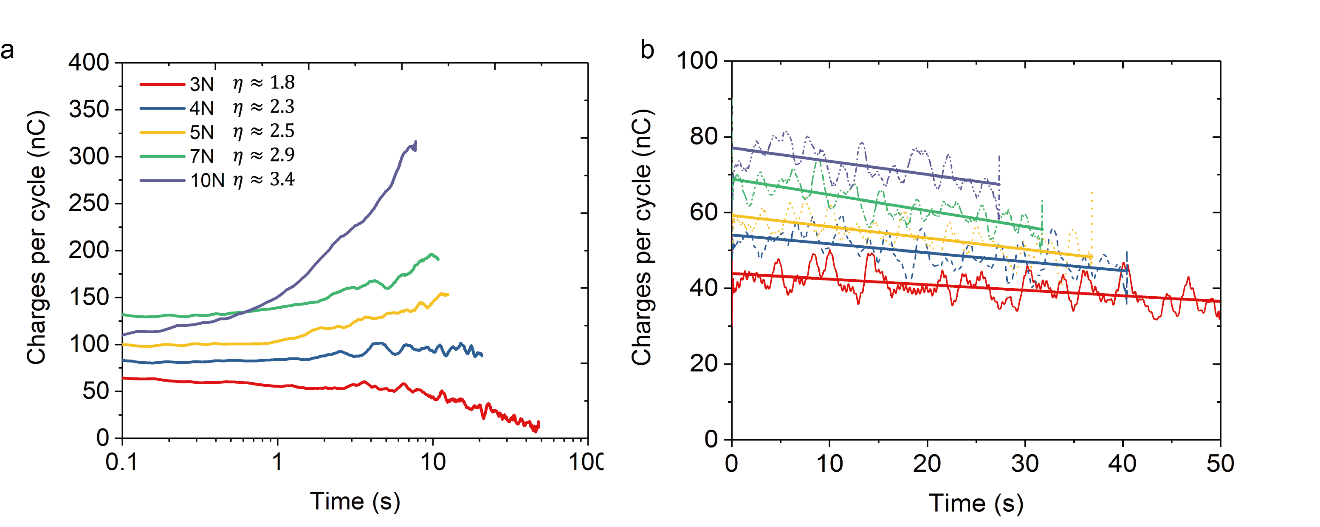


**Supplementary Figure 5**. The harvested charges per cycle using Bennet doubler **(a)** and full-wave rectifier **(b)** to directly charge a 4.7 nF capacitor. Different drive forces (from 3 N to 10 N) result in different capacitance variations thus different performance of the harvested charges. Only if the capacitance is higher than 2, i.e. $\text{C}_{\text{max}}>2\text{C}_{\text{m}\text{in}}$, an exponential occurs. Otherwise, the performance of the Bennet is similar or even worse than fullwave rectifiers. Source data are provided as a Source Data file.


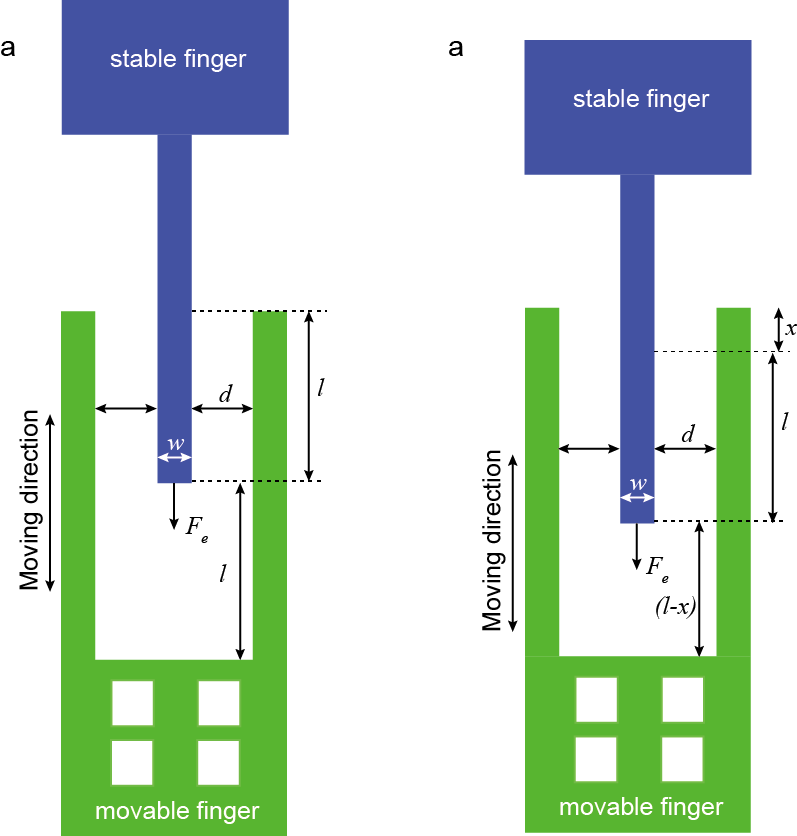


**Supplementary Figure 6**. Illustration of a pair of comb finger without **(a)** and with **(b)** a displacement.

**Supplementary Figure 7.** Simulated Electric fields at point A (anode) and point C (cathode) in Fig. 3b with three pairs of tips under different applied voltages. Source data are provided as a Source Data file.





**Supplementary Figure 8.** Long-term operation stability of the fixed plasma switch with triangular tips gap 7 μm. The ON/OFF voltage, i.e. the hysteresis loop, keeps almost constant within 3000 operation cycles. Source data are provided as a Source Data file.


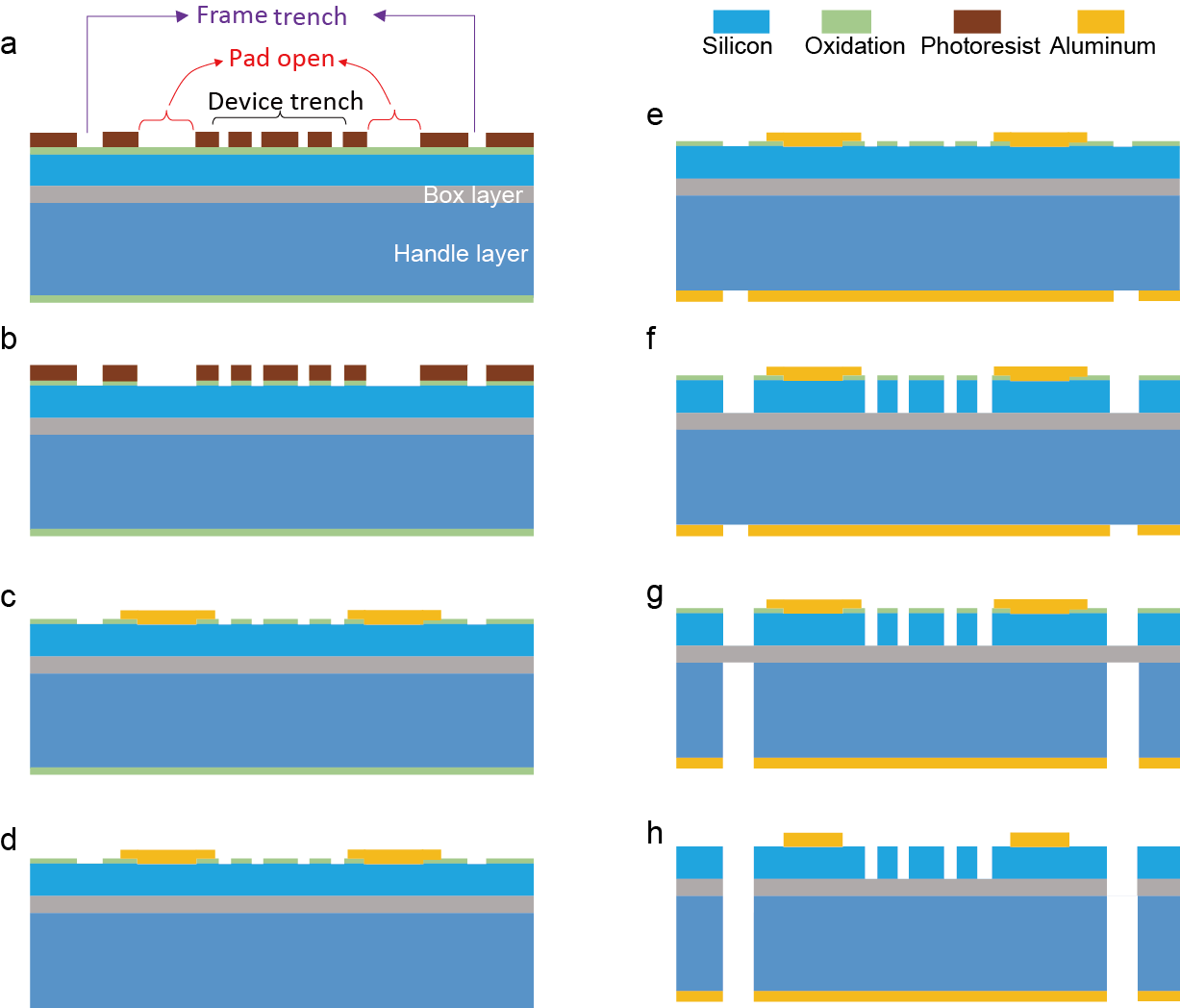


**Supplementary Figure 9.** Fabrication process of the MEMS switch.The fabrication of the switch (for both fixed-electrode and movable-electrode ones) is based on the dicing-free silicon-on-insulator (SOI) technique. The SOI wafer used in our fabrication has a 40 μm device layer, a 2 μm box layer and a 400 μm handle layer.

1. Photolithography for front-side DRIE and pads opening
2. Front DRIE for the pads opening
3. Remove photoresist and aluminum sputtering (1μm)
4. Remove the backside oxidation using buffered oxide etching
5. Backside aluminum sputtering (0.5μm)
6. Front DRIE etching until the box layer
7. Backside DRIE etching until the box layer
8. Vapour HF release


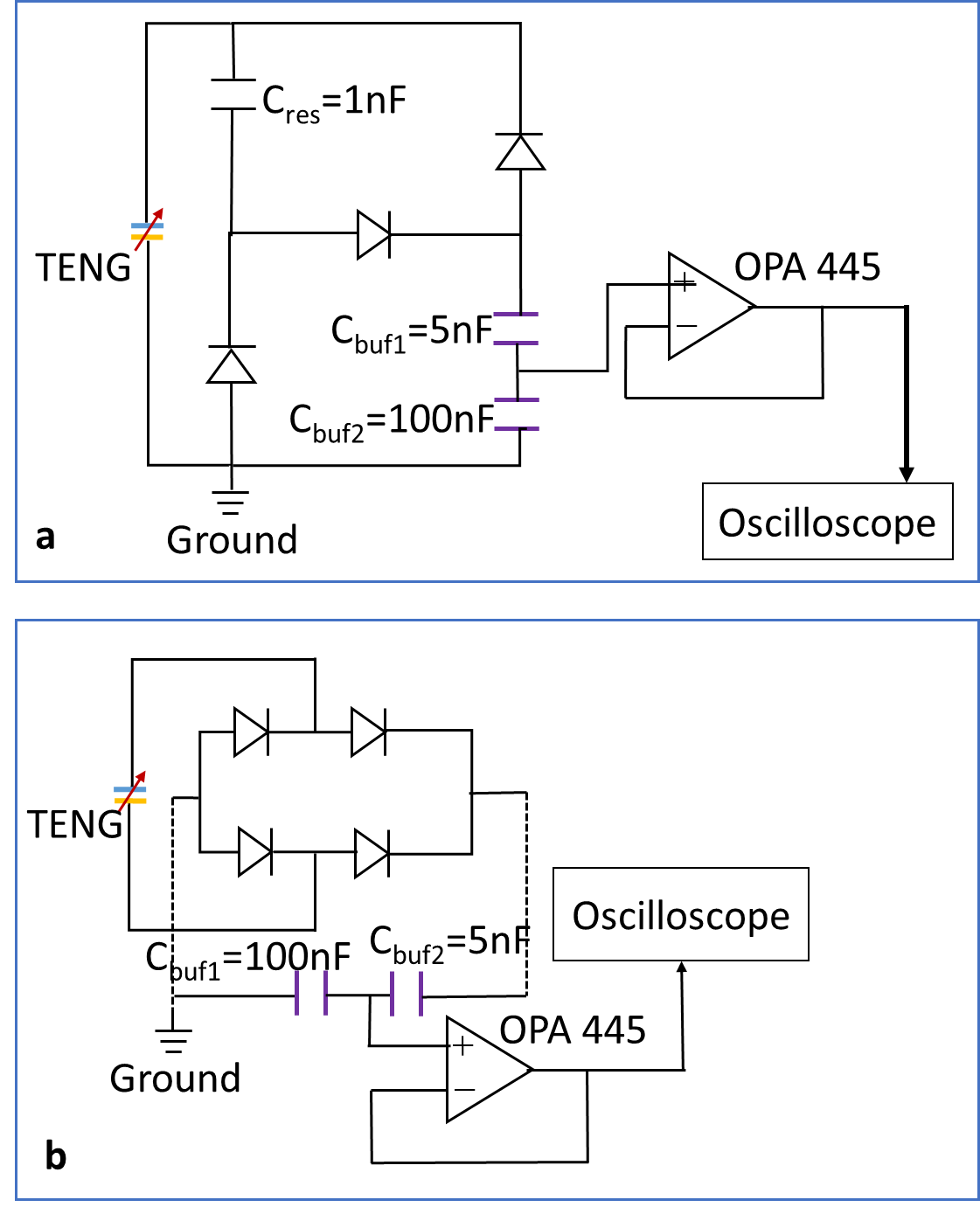


**Supplementary Figure 10.** Electrical measurement setup for the Bennet douber (a) and full-wave rectifier (b). Each diode in the circuits reports two diodes with 285 V inverse voltage in series. It is used to overcome the voltage limitation of the follower since two diodes can enlarge the reverse voltage from 285 V to 570 V. $\text{C}_{\text{res}}$ is a series combination of $\text{C}_{\text{res2}}\text{ }\text{=}\text{ }\text{100 nF}$ and $\text{C}_{\text{res1}}\text{ }\text{=}\text{ }\text{5 nF}$, thus the voltage applied on $\text{C}_{\text{res}}$ is calculated by: $\text{V}_{\text{C}_{\text{res}}}\text{=}\text{V}_{\text{osc}}\text{(1+(}{\text{C}_{\text{res2}}}/{\text{C}_{\text{res1}}}\text{))}$, where $\text{V}_{\text{osc}}$ is the voltage measured with the oscilloscope.

**Supplementary References**

1. Legtenberg, R., Groeneveld, A.W., & Elwenspoek, M. Comb-drive actuators for large displacements. *J. Micromech. Microeng*. **6**, 320 (1996).
2. Kisliuk, P. Electron emission at high fields due to positive ions. *J. Appl. Phys.* **30**, 51-5 (1959).
