# Supplementary material for: Employing a MEMS plasma switch for conditioning high-voltage kinetic energy harvesters
Source: Nat Commun. 2020 Jun 26;11:3221. doi: 10.1038/s41467-020-17019-5 (PMC7319968; doi:10.1038/s41467-020-17019-5)
Supplement: Supplementary file 3 — Description of Additional Supplementary Files [file 41467_2020_17019_MOESM3_ESM.docx]

Description of Additional Supplementary Files

File name: Supplementary Movie 1

Description : ON/OFF actuation of the plasma switch with fixed electrodes

File name: Supplementary Movie 2:

Description: A TENG conditioned by the proposed 2-stage circuit continuously drives a 3.3V watch
